# Cytosolic Entry of Shiga-Like Toxin A Chain from the Yeast Endoplasmic Reticulum Requires Catalytically Active Hrd1p

**DOI:** 10.1371/journal.pone.0041119

**Published:** 2012-07-19

**Authors:** Shuyu Li, Robert A. Spooner, Randolph Y. Hampton, J. Michael Lord, Lynne M. Roberts

**Affiliations:** 1 School of Life Sciences, University of Warwick, Coventry, United Kingdom; 2 Section of Cell and Developmental Biology, Division of Biology, University of California San Diego, La Jolla, California, United States of America; University of Pittsburgh, United States of America

## Abstract

**Background:**

*Escherichia coli* Shiga-like toxin 1 normally traffics to the endoplasmic reticulum (ER) in sensitive mammalian cells from where the catalytic A chain (SLTxA1) dislocates to the cytosol to inactivate ribosomes. Currently, no molecular details of the dislocation process are available. To investigate the mechanism of the dislocation step we expressed SLTxA1 in the ER of *Saccharomyces cerevisiae.*

**Methodology and Principal Findings:**

Using a combination of growth studies and biochemical tracking in yeast knock-out strains we show that SLTxA1 follows an ER-associated degradation (ERAD) pathway to enter the cytosol in a step mediated by the transmembrane Hrd1p ubiquitin ligase complex. ER-to-cytosol dislocation of the bulk population of SLTxA1 requires Cdc48p and its ubiquitin-handling co-factor Npl4p, and this population of toxin is terminally dispatched by proteasomal degradation. A small sub-population of SLTxA1 uncouples from this classical ERAD pathway and recovers catalytic activity in the cytosol. The pathway that leads to toxicity is also Hrd1p-dependent but, unlike that for the related ricin A chain toxin, SLTxA1 dislocation *does* require the catalytic cysteine of Hrd1p. However it does not depend on canonical ubiquitylation since toxin variants lacking endogenous lysyl residues also utilize this pathway, and furthermore there is no requirement for a number of Cdc48p co-factors.

**Conclusions and Significance:**

The fraction of SLTxA1 that disengages from the ERAD pathway thus does so upstream of Cdc48p interactions and downstream of Hrd1p interactions, in a step that possibly involves de-ubiquitylation. Mechanistically therefore, the dislocation of this toxin is quite distinct from that of conventional ERAD substrates that are normally degraded, *and* the toxins partially characterised to date that do not require the catalytic cysteine of the major Hrd1p component of the dislocation apparatus.

## Introduction

Endoplasmic reticulum (ER) associated protein degradation (ERAD) comprises multiple disposal pathways that recognize and remove terminally misfolded and orphan proteins from the ER membrane and lumen. Removal (dislocation) normally requires specific membrane-bound ubiquitin ligase complexes that polyubiquitylate the target substrates [1], [2], providing tags for an ERAD-enabled cytosolic AAA-ATPase p97/Cdc48p complex extraction motor [3], [4], [5], [6] and, after limited deubiquitylation [7], [8], for subsequent binding to proteasomes [9], [10]. Following dislocation, these ERAD substrates become de-glycosylated (if appropriate), de-ubiquitylated and are then destroyed by proteasomal degradation [11].

A number of proteins are thought to utilize ERAD components to reach the cytosol but then disengage from canonical ERAD pathways and so avoid degradation in the proteasome core. Principal amongst these are the enzymatic A chains of some plant and bacterial toxins which traffic in vesicular carriers from the cell surface of target mammalian cells to the ER lumen, where the toxic A chain and cell-binding B chain(s) are separated [12], [13], [14]. The A chains are then thought to undergo structural changes that allow them to be recognized by ER quality control systems as dislocatable substrates: proposed mechanisms include ER membrane interactions of a hydrophobic domain of ricin A chain previously masked in the holotoxin by ricin B chain [15]; chaperone-mediated unfolding of cholera toxin A chain [16]; and thermal instability of the free A chains [17], [18]. After crossing the ER membrane, the A chains utilise cytosolic chaperones to refold to a catalytic conformation [19], [20] that allows modification of their cytosolic targets.

The yeast viral AB toxin K28 also dislocates from the yeast ER and subsequently its A chain recovers catalytic activity in the cytosol [21]. In addition, ER dislocation has been proposed for the human hepatitis E virus ORF2 protein [22], hepatitis B virus precore protein [23], luciferases taken up by macropinocytosis into dendritic cells [24] and for a cytosolic pool of functional calreticulin, a protein normally regarded as an ER resident [25], [26]. Uncoupling from the final destructive stages of ERAD may therefore be a normal cellular pathway for which only a few substrates have been identified to date.

ER-dislocating toxins are powerful probes of the ER dislocation and ERAD-uncoupling pathways, as their toxicity towards eukaryotic cells can be exploited to identify which ERAD components are required and which are not. This has been exemplified by study of the plant toxin ricin, whose A chain (RTA) dislocates from the ER. Although this process for RTA is relatively uncharacterized in mammalian cells, we have begun to define ER pre-dislocation, dislocation and cytosolic post-dislocation interactions by expressing RTA in the yeast ER lumen [27]. Following dislocation, a significant proportion of RTA is degraded by an unidentified cytosolic protease (not the proteasome). However the remainder folds to an active conformation that inactivates the ribosomal targets [28], reducing yeast protein synthesis activity and severely inhibiting growth. Mutant yeast strains defective in dislocation stabilised RTA in the ER lumen and also grew well, allowing us to identify cellular components required for the dislocation of RTA. Following its delivery to the ER, RTA cycles to the Golgi before Cdc48p-independent dislocation via the Hrd1/Hrd3/Der1 E3 ubiquitin ligase complex in a manner that does not require the E3 ubiquitin ligase activity of Hrd1p [27]. A recent report showing that the response of mammalian cells to ricin challenge is sensitive to manipulated expression levels of the SEL1L regulator of the mammalian HRD dislocation complex [29] suggests that these findings in yeast are relevant to RTA dislocation in mammalian cells. Furthermore, in mammalian cells, the E3 ubiquitin ligases HRD1 and gp78 promote the dislocation of the A1 chain of cholera toxin [30].

Here we investigate the fate of the toxic A chain of Shiga-like toxin (SLTx) delivered to the yeast ER lumen by the Kar2p signal peptide. SLTx is an AB_5_ toxin produced by enterohemorrhagic strains of *E. coli*, that is essentially identical to Shiga toxin (STx) produced by *Shigella dysenteriae*. On susceptible mammalian cells, the STxB/SLTxB chain pentamer binds its glycolipid receptor and the toxin is endocytosed to the ER lumen. Here the furin-processed toxic A chain [31] (the STxA1/SLTxA1 fragment) is liberated from the holotoxin and dislocated to the cytosol where it folds to an active conformation. Like RTA, cytosolic STxA1/SLTxA1 specifically depurinates the large ribosomal subunit to inactivate ribosomes [32]. Whilst multiple molecular details are known for STx/SLTx trafficking requirements in mammalian cells [33], very few are known for pre-dislocation, dislocation and post-dislocation events, beyond interactions of STx with a pre-assembled ER luminal protein complex containing the chaperones HEDJ, BiP and GRP94 associated with the Sec61 translocon core unit [34], [35], and the importance of the C-terminal hydrophobic region of SLTxA1 [36] that may interact with the ER membrane [37], [38].

We report here the expression of SLTxA1 in the yeast ER and a dissection of its requirements for dislocation. Like RTA, SLTxA1 dislocates in a Hrd1p-dependent manner, but unlike RTA, the fraction that recovers catalytic activity *does* require the catalytic cysteine of the core dislocon component Hrd1p. Post-dislocation, a proportion of SLTxA1 evades the cytosolic Cdc48p complex and subsequent targeting to the proteasome core, allowing an uncoupling from ERAD and the expression of toxin activity in the cytosol.

## Results

### ER-imported SLTxA1 is O-mannosylated

**Figure 1:**
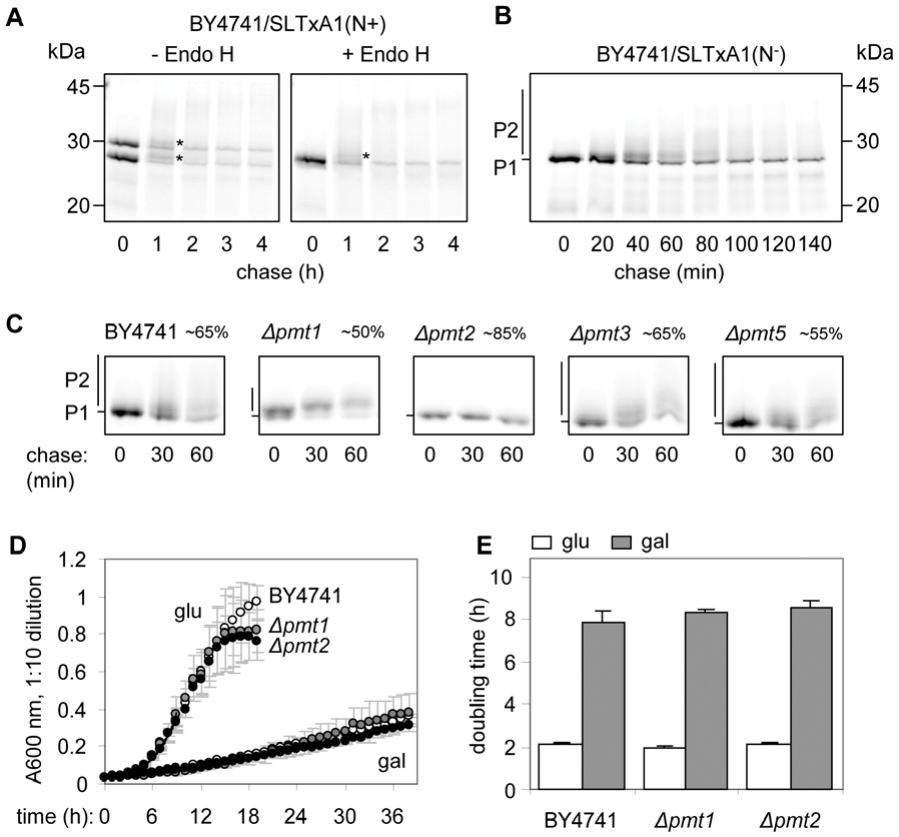
ER-imported SLTxA1 is *N*-glycosylated and further modified. **A.** Left hand panel; SLTxA1 (N+, with a non-mutated *N*-glycosylation sequon) was immunoprecipitated from detergent soluble extracts of wild-type cells expressing SLTxA1(N+), prepared after a pulse of [^35^S]-methionine/cysteine with or without a subsequent chase of unlabelled amino acids for the times indicated. Right hand panel; samples were prepared as in the left-hand panel, but subsequently treated with EndoH. Samples were analysed by SDS-PAGE and fluorography. Positions of migrations of size standards are marked on the left. * and slow migrating smears, EndoH resistant SLTxA1. **B.** Pulse-chase kinetics of SLTxA1 (N^−^, with a mutated *N*-glycosylation sequon), with samples treated as in **A**. P1, ∼27.5 kDa major band; P2, ∼28 kDa indistinct band and higher molecular weight smear. Positions of migrations of size standards are marked on the right. **C.** Pulse-chase kinetics of SLTxA1(N^−^) in wild-type yeast BY4741 and in strains deleted of individual protein *O*-mannosyl transferases. Fluorograms were quantified using TotalLab software, and the approximate levels of protein (rounded to the nearest 5%) in the one-hour chase are given, relative to the pulse. **D.** Growth of wild-type, *Δpmt1* and *Δpmt2* yeast carrying the SLTxA1(N^−^) plasmid in non-inducing (glucose) and inducing (galactose) liquid growth medium. Means of three independent colonies grown in parallel are given, bars = 1 S.D. **E.** Doubling times (+1 S.D.) derived from the exponential phase of **D**.

Pulse-labeled ER-directed SLTxA1 migrated as two bands in SDS-PAGE, the upper (∼30 kDa) representing a singly *N*-glycosylated form since it was sensitive to EndoH digestion (Fig. 1A). Upon chase, each of the two forms was further modified in an EndoH-resistant manner (asterisks and smears of slow migrating species, Fig. 1A), and over time, SLTxA1 disappeared, suggesting its degradation. SLTx holotoxin is a bacterial protein and so is not normally *N*-glycosylated, and there was no detectable *N*-glycosylation of this protein seen after mammalian cell challenge with holotoxin (Fig. S1). Thus the *N*-glycosylation observed here was likely generated at a cryptic glycosylation sequon after direct ER expression of its A1 chain in a eukaryotic organism. Alteration of the single predicted *N*-glycosylation site of SLTxA1 (substituting glutamine for asparagine 83) to generate SLTxA1(N^−^) resulted in expression of a single species (P1, ∼27.5 kDa, Fig. 1B) which was modified over time to higher molecular weight forms (P2, comprising an indistinct blurred band at ∼28 kDa and higher molecular weight smear, Fig. 1B).

These additional modifications are suggestive of extensive *O*-mannosylation. We therefore examined the fate of SLTxA1(N^−^) in single gene knockouts of members of the PMT family, the ER-localised protein *O*-mannosyl transferases that catalyse the transfer of mannose from dolichyl phosphate-activated mannose to available seryl or threonyl residue acceptor sites on target proteins [39]. Pmt1p and Pmt2p normally form a heterodimeric complex, as do Pmt3p and Pmt5p, but deletion of individual PMT genes results in production of alternative heterodimers [40], [41]. Individual deletion of PMT3 or PMT5 made little difference to SLTxA1(N^−^) phenotypes (Fig. 1C). However, the remaining PMT heterodimers after PMT1 deletion appeared to *O*-mannosylate SLTxA1(N^−^) with altered efficiency or specificity, and we saw an absolute requirement for Pmt2p, since in its absence SLTxA1(N^−^) was expressed as a single species (Fig. 1C). Accurate quantitation of smeared bands is difficult, but in the absence of *O*-mannosylation, SLTxA1(N^−^) appeared to have increased stability (*Δpmt2*, Fig. 1C), suggesting that the time-dependent disappearance of SLTxA1(N^−^) from the ER occurred mostly through loss of the *O*-mannosylated forms.

SLTxA1 expression in the yeast ER is followed by dislocation, causing a degree of ribosome modification and therefore reduced protein synthesis and a reduced cell growth rate [36]. The consequences of altering *O*-mannosylation patterns were examined by spotting dilutions of wild-type and PMT deletion strains expressing SLTxA1(N^−^) on to non-inducing (glucose) and inducing (galactose) nutrient plates, and monitoring subsequent growth. We could see no differences between the strains (not shown), so we therefore measured growth in liquid cultures (Fig. 1D), and determined the doubling times of the cultures from the exponential phase of growth (Fig. 1E). After induction of expression in galactose medium, there was no discernible difference in the growth rates of wild-type cells (expressing P1and P2 smears), *Δpmt1* cells (expressing P1 and lower molecular weight P2 smears) or *Δpmt*2 cells (expressing only the non-mannosylated P1 protein). Since the toxicity of SLTxA1 in *Δpmt2* cells was identical to that observed in the wild type strain, we conclude that the toxic fraction of cytosolic SLTxA1(N^−^) is likely to be derived from the ER population that is *not O*-mannosylated, supporting the view that *O*-mannosylated proteins may be more tightly coupled to the degradation pathways [42].

### The bulk of SLTxA1(N−) behaves as a classical ERAD substrate

**Figure 2:**
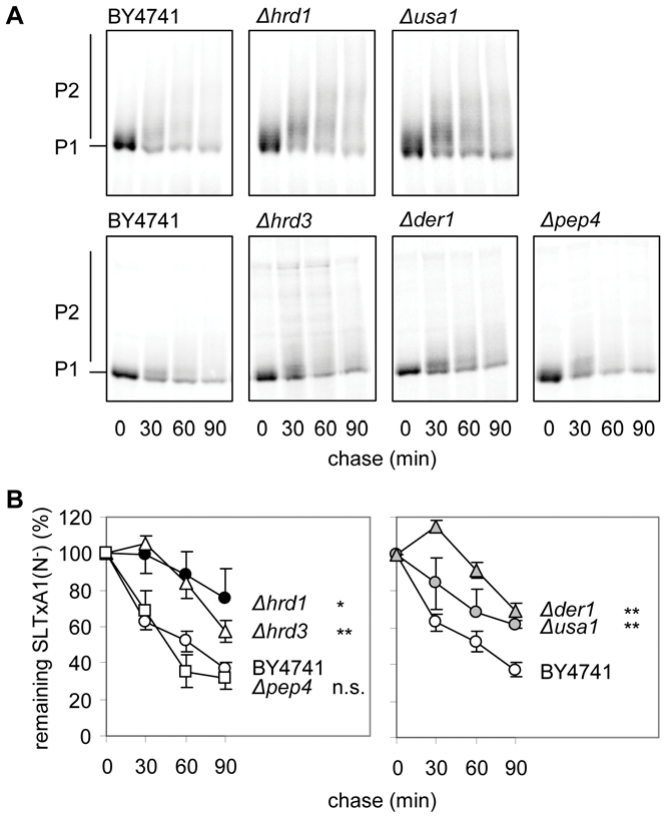
SLTxA1 is dislocated by the Hrd1p complex. **A.** Pulse-chase analysis of SLTxA1(N*^−^*) in yeast strains null for the some members of the Hrd1p dislocation complex (*Δhrd1*, *Δusa1*) compared with the congenic wt BY4741 performed at the same time (upper panels) and in strains null for other members of the Hrd1p dislocation complex (*Δhrd3*, *Δder1*) and activation of vacuoalar proteinases (*Δpep4*), again compared with BY4741 performed in parallel (lower panels). **B.** Quantitations of experiments performed as in **A** (n = 3; bars, +/−1 S.D. Overlapping error bars have been removed for clarity). Statistical comparisons with BY4741 were made at the 90 minute time-point, using non-paired two-tailed tests: n.s., * and **; non-significant, significant (p<0.05) and very significant (p<0.01), respectively.

Extended ER-Golgi residence of SLTxA1(N^−^), as monitored by increased *O*-mannosylation (Fig. 2A) and by densitometric measurement after phosphorimaging of immunoprecipitated protein (Fig. 2B) resulted from individual deletion of the core Hrd1p E3 ubiquitin ligase [43], its Hrd3p [44] and Usa1p [45] regulators, or the Der1p adaptor that both recruits the cytosolic Cdc48p complex [46] and permits Hrd1-dependent dislocation of ERAD-L substrates with misfolded luminal domains [1], [47]. SLTxA1(N^−^) did not appear to access the vacuole, since its fate was unaltered in a strain lacking Pep4p, an enzyme required for the maturation of vacuolar proteinases (Fig. 2A, B). Thus, SLTxA1(N^−^) expressed in the ER appears to be dislocated to the cytosol via the ERAD-associated Hrd1p dislocation complex.

**Figure 3:**
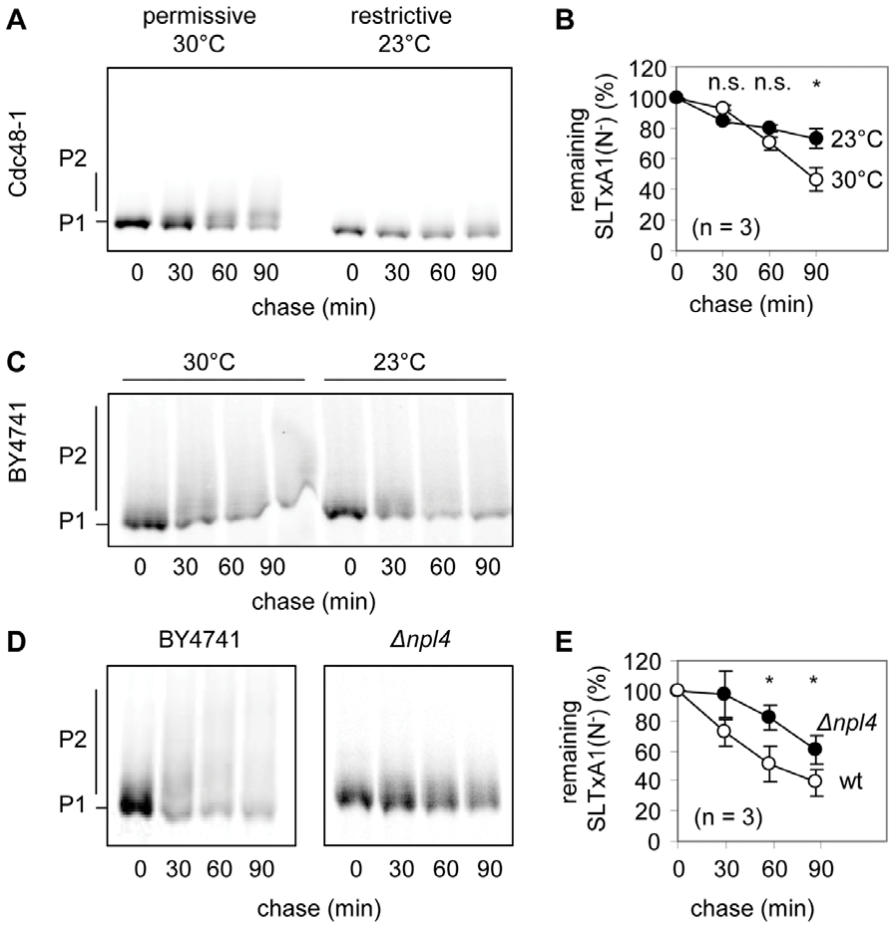
The bulk population of SLTxA1 is extracted by Cdc48p and its co-factor Npl4. **A.** Pulse chase analysis of SLTxA1(N^−^) in the cold-sensitive Cdc48-1 yeast strain at the permissive (30°C) and restrictive (23°C) temperatures for growth. **B.** Quantitation of three independent experiments performed as in **A. C.** Pulse chase analysis of SLTxA1(N^−^) in the WT yeast strain BY4741 at 30°C and 23°C. **D.** Pulse chase analysis of SLTxA1(N^−^) in WT and *Δnpl4* cells. **E.** Quantitation of three independent experiments performed as in **D**. In **B** and **D**, statistical comparisons were made at the time-points indicated using two-tailed tests: n.s., non-significant; *, significant (p<0.05).

In a conditional mutant (Cdc48-1) of CDC48, at the restrictive temperature that inactivates Cdc48p, we noted stabilization of SLTxA1(N^−^) suggesting a role in ER dislocation of the toxin subunit (Fig. 3A, B). At this lowered temperature, we also noted a delay in acquisition of elaborated *O*-mannose structures (Fig. 3A), that is not simply a consequence of reduced temperature (Fig. 3C). This suggests that there may also be a requirement for CDC48 functions in ER-Golgi cycling, perhaps related to its membrane fusion activities [48], [49]. The toxin subunit was also stabilized in a yeast strain lacking Npl4p, a substrate recruiting co-factor of Cdc48p that adapts the complex for ERAD (Fig. 3D, E). Taken together, these data suggest ER extraction of SLTxA1(N^−^) by an ERAD-enabled Cdc48p complex. Consistent with this, we found the bulk population of SLTxA1 was polyubiquitylated, with fewer lower molecular weight forms visible in the presence of a proteasome inhibitor (Fig. S2).

**Figure 4:**
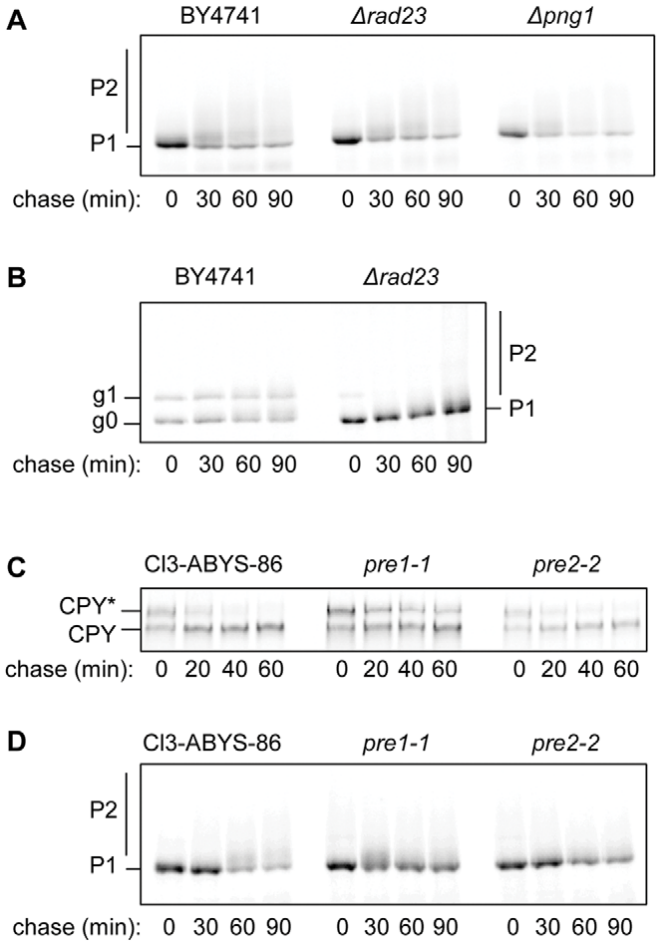
The bulk population of SLTxA1 is destroyed by proteasomal proteolysis. **A.** Pulse-chase analysis of SLTxA1(N^−^) in yeast strains null for the Cdc48 to proteasome shuttling factors Rad23p and Png1p and in BY4741 performed at the same time. **B.** Pulse-chase analysis of SLTxA1(N+) in WT and *Δrad23* yeast. **C.** Pulse-chase analysis of CPY* (upper band) in *pre1-1* and *pre2-2* proteasomal degradation mutants and the congenic wild-type C13-ABYS-86. These strains also express wt CPY (lower band). **D.** Pulse-chase analysis of SLTxA1(N^−^) in *pre1-1* and *pre2-2* and wild-type C13-ABYS-86.

As expected for a protein lacking *N*-glycans, there was no discernable role in SLTxA1(N^−^) dislocation for the cytosolic Rad23p/Png1p proteasomal receptor complex [50] that removes *N*-glycans from ERAD substrates prior to their proteasomal destruction (Fig. 4A). This was in marked contrast to the *N*-glycosylated version, SLTxA1(N^+^), which was very strongly stabilized and deglycosylated in the absence of Rad23p (Fig. 4B). The ultimate fate of the bulk population of SLTxA1(N^−^) was degradation within proteasomes, since it was stabilized like the authentic ERAD substrate CPY* (Fig. 4C) in the *pre1-1* yeast strain (Fig. 4D) that is mutated in the catalytic β4 subunit of the 20S proteasome [51]. In addition we noted increased stability of SLTxA1(N^−^) to a lesser extent in the *pre2-2* yeast strain that is mutated in the β5 subunit of the 20S proteasome (Fig. 4D).

### Toxicity of SLTxA1(N−) requires the E3 ubiquitin ligase activity of Hrd1p

Biochemical analyses such as the pulse-chase experiments above show that SLTxA1(N^−^) behaves as an ERAD substrate that is ultimately degraded by the proteasome. However, since its expression is toxic to yeast cells [36], this implies that a small proportion, not visible by conventional biochemistry, must evade the proteasome to recover activity in the cytosol. The biochemical approach is therefore uninformative in charting the pathway taken by the fraction that recovers toxic activity. In contrast, drop tests reveal functional activity of the toxin in the yeast cytosol and so *can* be used to elucidate the mechanisms by which some proteins recover a native conformation following dislocation [27].

**Figure 5:**
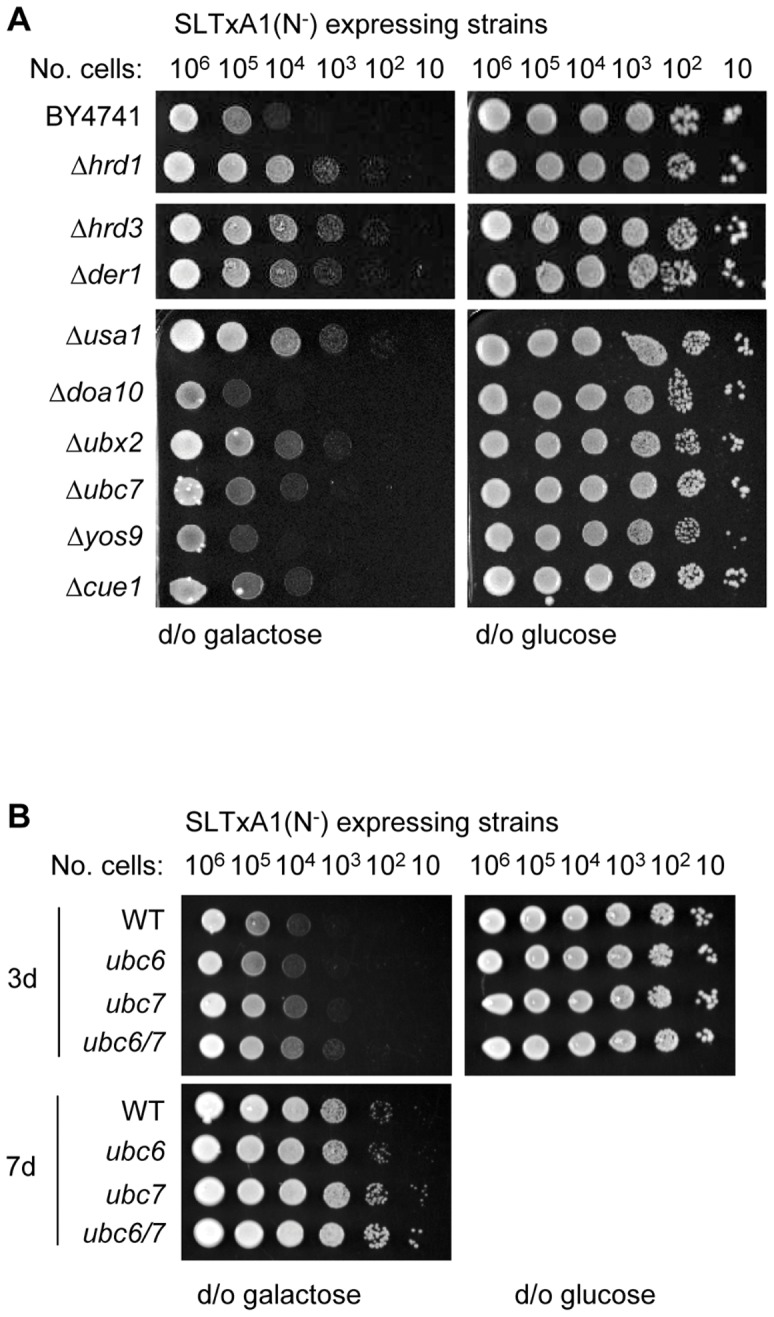
The fraction of SLTxA1(N^−^) that recovers activity in the cytosol is dislocated by the Hrd1 complex. **A.** Ten-fold dilutions of WT (BY1747) and derivative strains null for genes associated with ER dislocation were transformed with a plasmid expressing SLTxA1(N^−^) and applied to inducing (d/o galactose) and non-inducing (d/o glucose) plates and grown for 3 d. **B.** Wt, ubc6, ubc7 and ubc6–ubc7 expressing SLTxA1(N^−^) strains were diluted as in **A** and grown for the indicated times.

Yeast strains lacking Hrd1p, Hrd3p, Der1p and Usa1p had a growth advantage on galactose medium compared to the congenic wild-type BY4741 (Fig. 5A): in addition, growth tests also identified intermediate requirements for Ubx2p, which recruits the cytosolic Cdc48 complex to the Hrd1 complex [52], [53] but not for the E2-ubiquitin ligase conjugating enzyme Ubc7p and its activating partner, the membrane–integral Cue1p [54], [55]. A *ubc6-ubc7* strain [56] had a slight growth advantage over wild-type cells expressing SLTxA1(N^−^) (Figure 5B), that was only clearly apparent after extended growth (7 days, lower panel). This suggests that although both these ubiquitin-conjugating enzymes are required for optimal toxicity of SLTxA1(N^−^) toxicity, the roles of each are minor.

As expected for a non *N*-glycosylated substrate, there was no obvious role for Yos9p (Fig. 5A), which binds *N*-glycans that have been modified by Htm1p and delivers substrates bearing these to the Hrd1 complex [57], [58], [59], [60], [61]. Nor was there an obvious role identified for Doa10p, which recognizes lesions in the cytosolic domains of transmembrane ERAD-C substrates [1].

**Figure 6:**
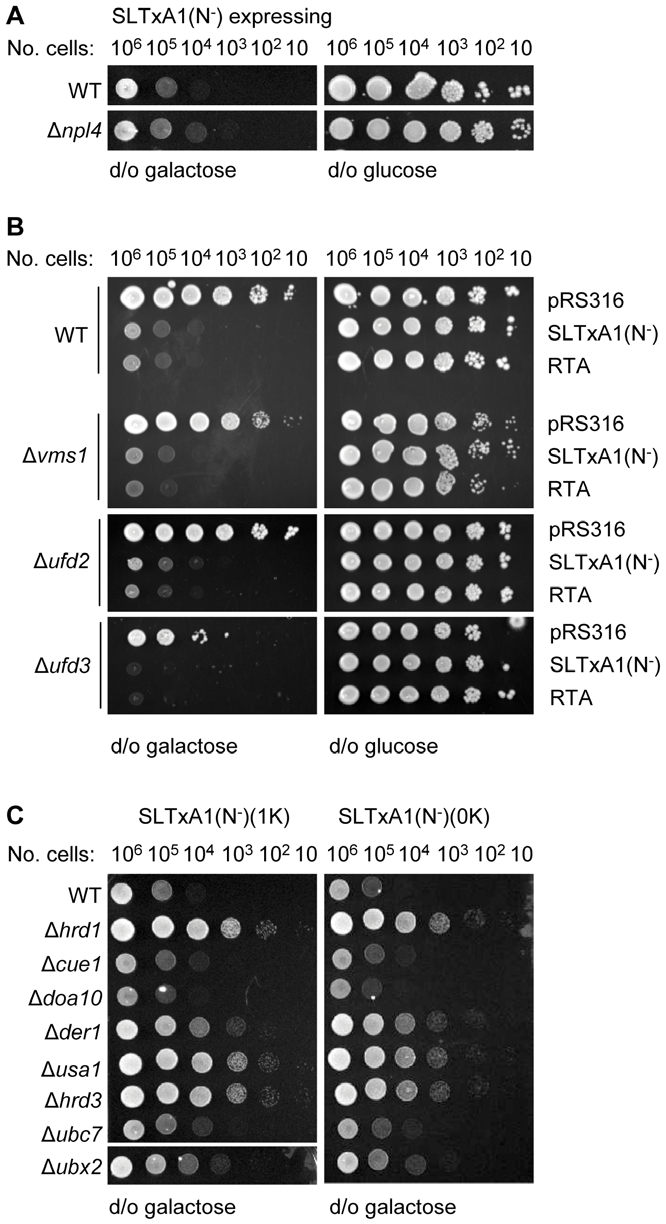
Canonical ubiquitylation is not required for dislocation of the toxic fraction of SLTxA1. **A.** Growth of WT(BY4741) and *Δnpl4* cells expressing SLTxA1(N^−^) on inducing (d/o galactose) and non-inducing (d/o glucose) plates. **B.** Growth of WT, *ΔVms1*, *ΔUfd2* and *ΔUfd3* yeast strains transformed with vector (pRS316) or expressing RTA or SLTxA1(N^−^) on inducing (left) and non-inducing (right) plates **C.** Viabilities of WT and the indicated mutant strains expressing SLTxA1(N^−^) variants lacking one (1K) or both (0K) endogenous lysyl residues on inducing (galactose) media.

Despite the partial stabilization of SLTxA1(N^−^) in *Δnpl4* yeast revealed by pulse-chase analysis (Fig. 3D), such cells displayed no obvious growth advantage in drop tests over wild-type cells expressing the toxin subunit (Fig. 6A). We could therefore find no clear role for Npl4 (a co-factor that adapts the Cdc48p complex for binding ubiquitylated ERAD substrates) in permitting dislocation of a catalytically active SLTxA1(N^−^). To examine this further we tested a number of other Cdc48 co-factors with roles in regulating the ubiquitylation status of Cdc48 clients, in parallel testing RTA as a negative control that avoids Cdc48p and Npl4p interactions [27] (Fig. 6B). We could find no obvious role for Vms1p, a protein with a role in release of ubiquitylated ERAD substrates from Cdc48 complexes [62]. Furthermore we could not discern a function in SLTxA1 toxicity for either of two other Cdc48 co-factors; the ubiquitin-chain extending enzyme Ufd2p and its antagonist Ufd3p which can bind simultaneously with the deubiquitylase Otu1p [63]. Taken together, these observations suggest that despite the bulk population of SLTxA1(N^−^) being extracted in a ubiquitin-dependent manner by a Cdc48 complex (Fig. 3) the small subpopulation that is destined to recover catalytic activity in the cytosol might be removed from the ER in a ubiquitin-independent manner, and thus may avoid Cdc48 interactions in a manner similar to RTA [27]. Consistent with this idea, changing one or both of the two endogenous lysines of SLTxA1 to arginine did not alter the requirement for Hrd1p or the other dislocation–associated proteins for an active toxin in the cytosol in drop tests (Fig. 6C). It appears that canonical ubiquitylation on internal lysines of SLTxA1(N^−^) is not essential for the restoration of activity after dislocation and that it is probably a non-ubiquitylated fraction of the toxin subunit that is better suited to recover activity in the cytosol.

**Figure 7:**
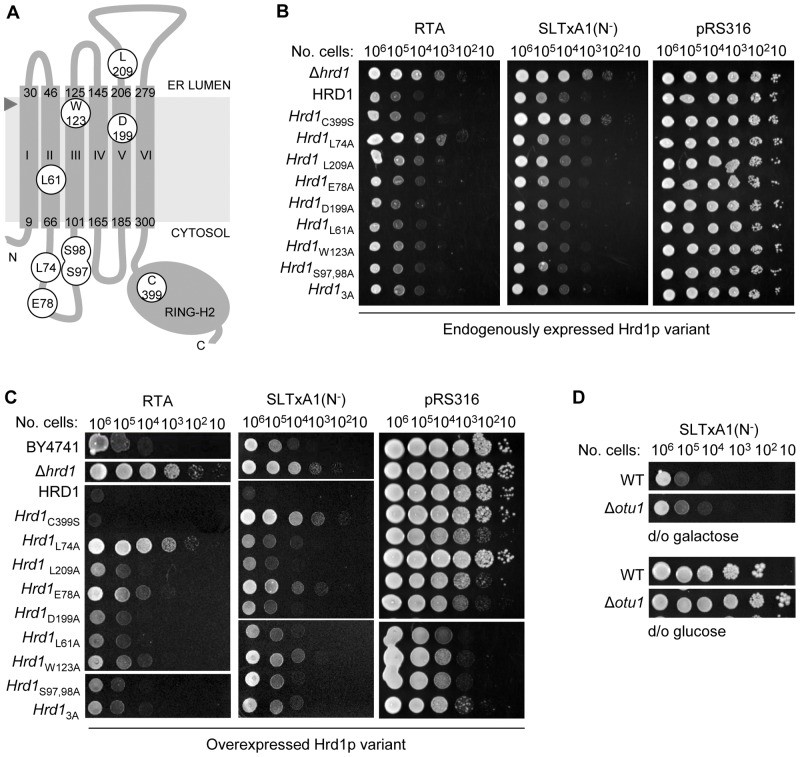
The toxic fraction of SLTxA1 requires particular residues of Hrd1p. **A.** Topological model of Hrd1p, based on transmembrane segment prediction by the TOPCONS predictor [78], marked (white filled rings) with positions of mutated residues important for E3 ubiquitin ligase activity of the RING-H2 domain [79] and ERAD-M substrate recognition [64]. This model differs in transmembrane definition (segments I–VI, residues 9–30, 46–66, 101–125, 145–165, 185–206, and 279–300 respectively, marked) and therefore relative position of these residues from that presented by Sato et al [64], but both are consistent with a proposed six transmembrane spanning model [79]. The arrowhead marks a potential signal peptide cleavage site between residues S27 and A28 that is predicted by the SignalP server [80]. **B.** RSY3011 (*Δhrd1*) yeast was transformed separately with yeast integrating plasmids expressing HRD1p or Hrd1p variants (3A refers to the previously described [64] triple mutant S97A, S98A, D199A) and each derivative strain was separately transformed with pRS316 (vector) or with pRS316 expressing either RTA or SLTxA1(N^−^). Serial dilutions of the strains were spotted on to (inducing) galactose plates and non-inducing glucose plates (not shown) and grown for 3 d. **C.** BY4741, a congenic *Δhrd1* strain and the latter separately transformed with plasmids expressing Hrd1p or mutated derivatives from the galactose promoter were selected and subsequently transformed separately with pRS316 (vector) or vector expressing RTA or SLTxA1(N^−^). Ten-fold dilutions were applied to inducing (galactose) and non-inducing (not shown) plates and grown for 3 d. **D.** Viabilities of WT (BY4741) and *Δotu1* yeast expressing SLTxA1(N^−^) on inducing (left) and non-inducing (right) media.

Toxicity of the plant toxin RTA, when similarly targeted to the yeast ER, shows a requirement for structural features of the Hrd1p protein but *not* its catalytic E3 ubiquitin ligase activity. This permits ubiquitin-independent dislocation and a bypass of Cdc48p and the proteasome core [27]. To investigate the role of Hrd1p further, we constructed yeast strains that express Hrd1p or *hrd1p* mutants driven by the endogenous Hrd1 promoter, as previously described [64]. All the mutants have differential effects on the dislocation of ERAD-M mutants whose lesions occur in transmembrane segments but have no measurable consequences on the dislocation of characterized ERAD-L mutants. Fig. 7A provides a topological model of those Hrd1p residues that are implicated in ERAD-M. Der1p-dependence is a hallmark of ERAD-L dislocation and since RTA has only a partial dependence on Der1p for dislocation [27], then it may act in part as an ERAD-M substrate, consistent with the embedding of the carboxy-terminus of free RTA in the ER membrane after release from holotoxin [15].

In the absence of toxin expression, these yeast strains show no obvious growth defect on galactose (Fig. 7B, vector controls, right-hand panel). Expression of RTA in these strains confirms the lack of requirement for C399, the catalytic cysteine of Hrd1p [27], and indeed further identifies a requirement for L74, suggesting a role for ERAD-M in RTA dislocation (Fig. 7A). In contrast to RTA, the toxic fraction of SLTxA1(N^−^) *does* require C399 for dislocation, but has no obvious requirements for any previously identified ERAD-M associated Hrd1p amino-acids (Fig. 7B), pointing towards mechanistic differences in the selection of these two toxin substrates by the dislocon machinery.

Overexpression of Hrd1p overrides the normal requirements for other members of the dislocon [44], [65], [66], although there are variable associated growth defects (Fig. 7C, vector controls, right-hand panel). Under these conditions, it is important to note that the dramatic differences in the toxin requirements for C399 and L74 observed above were confirmed, even though overexpression of Hrd1p L74 alone confers a moderate growth disadvantage (Fig. 7C, right hand panel). In addition, we observed additional common minor roles for the ERAD-M associated residues E78 and W123 for both toxins that were evident despite the moderate to severe toxicity, respectively, of overexpressing these Hrd1p mutants in the absence of toxin (Fig. 7C).

For SLTxA1(N^−^), the need for Hrd1p residue C399, presumably to add ubiquitin to the fraction of toxin that recovers activity in the cytosol, was surprising given that earlier we showed no requirement for the Cdc48 co-factors Npl4p (Fig. 6A), Vms1p, Ufd2p and Ufd3p (Fig. 6B) or for either of the two lysine residues of the toxin subunit (Fig. 6C). It might therefore be reasonable to assume that the fraction of toxin that recovers activity becomes ubiquitylated. Since degradation is not the immediate fate of the toxic fraction (otherwise it would not be toxic as revealed by drop tests), it is possible that rapid de-ubiquitylation in the cytosol affords an opportunity to recover toxicity. However, we could find no clear role for the most obvious candidate for de-ubiquitylation, the Cdc48p-associated de-ubiquitylase Otu1p (Fig. 7D) which can rescue Cdc48 substrates from proteasomal targeting [63]. Since Npl4p, Vms1p, Ufd2p, Ufd3p and Otu1p are all dispensable for recovery of toxicity, any proposed de-ubiquitylation of SLTxA1(N^−^) would therefore be expected to occur upstream of any Cdc48 complex interactions.

## Discussion

In yeast, the bulk of ER-imported SLTxA1 is *O*-mannosylated in the ER, which may be a consequence of its relatively long ER residence (T_1/2_∼60 min, estimated from Fig. 1B) compared to RTA (T_1/2_∼20 min [27]). A number of models have been presented for the role of Pmt2p-dependent *O*-mannosylation in ERAD. For mutant α-factor precursor, *O*-mannosylation slightly stabilizes this protein, suggesting a minor role in protecting substrates from ERAD by interfering with dislocation [67]. However for the misfolded substrate HA-Gas1*p, Pmt2p-dependent *O*-mannosylation is important for proteasomal degradation, and in the absence of this modification the substrate is primarily degraded in vacuoles [42]. SLTxA1(N^−^) shows considerable Pmt2p-stimulated *O*-mannosylation that is enhanced by the absence of the Hrd1p, Hrd3p, Usa1p and Der1p members of the Hrd1 ubiquitin ligase dislocation complex, but we see no evidence for a switching of the degradative compartment between the proteasome and the vacuole. Instead *O*-mannosylation may maintain solubility of SLTxA1 in the ER, as proposed for the ERAD substrates mutant prepro-α-factor and a pro-region-deleted derivative of aspartic proteinase-I [68]. The toxic fraction of SLTxA1 appears to be derived from the non-*O*-mannosylated forms, but increasing the proportion of these (in a PMT2 null strain) does not increase toxicity, suggesting that there are limiting factors in the yeast ER for dislocation and subsequent recovery of activity. In mammalian cells, ER trafficking of STx/SLTx appears to be efficient, since HRP-conjugated STx can be visualized in the ER by electronmicroscopy [69] and Cy-2 labelled SLTx can be visualized in the ER by fluorescence microscopy [70]. However, the dislocation frequency of the activated A1 chain is very low [71], suggesting that in mammalian cells also, there is an ER bottleneck for pre-dislocation events or dislocation itself.

There are two broad populations of ER-imported SLTxA1(N^−^); a major non-toxic fraction and a critical fraction that recovers activity in the cytosol and is responsible for toxicity and a resulting growth defect. We therefore used pulse-chase analysis to define the behavior of the bulk population of SLTxA1(N^−^) and used growth studies (drop tests) to describe the fate of the fraction of toxin that is destined to recover activity. Accurate quantitation of smeared bands after pulse-chase analysis is challenging, so stabilization in the ER was also judged visually by noting increased *O*-mannosylation of the toxin subunit. For the growth studies, a number of strains were unsuitable because growth would need to be performed at the restrictive temperature for growth (e.g. the cold-sensitive CDC48 mutant). Nevertheless, taking the data as a whole, we find that the bulk behavior of SLTxA1 is consistent with that of an authentic ERAD substrate that is extracted via the Hrd1/Hrd3/Der1 complex by Npl4p-adapted Cdc48p and which is terminally dispatched by the proteasome.

The fraction of the SLTxA1(N^−^) population that is destined to recover activity is also dislocated via a Hrd1p-dependent mechanism. A *hrd1* mutant previously characterized as specific for ERAD-M substrates, with no measurable effects on ERAD-L substrates [64], is partly defective in dislocation of the toxic sub-population of RTA, a protein that alters conformation in the presence of negatively charged lipids [72], and which embeds its hydrophobic C terminus into microsomal membranes [15]. The C-terminal region of SLTxA1 also contains a relatively hydrophobic stretch of amino acids that is important for cytotoxicity [36], and peptides based on this region interact with lipid membranes at low pH, possibly inserting at neutral pH [38], which may allow the toxin subunit to be perceived as ‘misfolded’ by ER quality control surveillance. However, effects of ERAD-M specific *hrd1* mutants on SLTxA1(N^−^) dislocation are only apparent following their overexpression, so our results suggest only a minor physiological role for this potential membrane piercing. It is clear that the two toxins negotiate dislocation idiosyncratically: although Hrd1p residues E78 and W123 play a minor role for dislocation of both toxin subunits, Hrd1p L74 is strongly required for RTA dislocation but has no obvious role for SLTxA1.

Cdc48 acts as a nexus for converging ERAD pathways that then targets substrates for proteasomal destruction [73]. However, RTA can uncouple from ERAD by dislocating in a ubiquitin-independent manner, thereby avoiding interactions with Cdc48p and subsequent proteasomal degradation [27]. Similarly, the K28 viral killer toxin dislocates without being ubiquitylated and without assistance from Cdc48p and its Npl4p and Ufd1p co-factors, and is not degraded by the proteasomal core [21]. Curiously though, the catalytic cysteine of Hrd1p *is* apparently required for the dislocation of the toxic fraction of SLTxA1, suggesting that this population is also extracted as a ubiquitylated protein. Presumably, such a modified polypeptide would need to be stripped of polyubiquitin chains to allow refolding to a functional conformation, which would suggest the intervention of a deubiquitylase. Since recovery of toxin activity did not depend on a battery of Cdc48p co-factors (the substrate recruiting Npl4p, the E4 ubiquitin chain extending factor Ufd2 and its competing Ufd3p antagonist, the de-ubiquitylase Otu1p and the substrate release factor Vms1p) such de-ubiquitylation would have to occur upstream of Cdc48 interactions. In mammalian cells, SLTx dislocation and toxicity are not influenced by chemical perturbation of the ubiquitin-proteasome system by eeyarestatin 1 (an inhibitor of p97 associated activities) or clasto-Lactacystin ß-lactone (an inhibitor of the proteasomal proteolytic activities) [74], again suggesting that the fraction of ER-delivered toxin that recovers activity in the cytosol does not interact with p97/VCP, the mammalian equivalent of Cdc48p. It appears that avoidance of Cdc48p interactions may be a common strategy for proteins that dislocate and avoid proteasomal proteolysis, but it remains unclear how a small fraction of SLTxA1 can perform this and whether an early de-ubiquitylation reaction of a type not normally sanctioned for ERAD substrates is involved. Furthermore, it is also not obvious why SLTxA1 requires the catalytic cysteine residue of Hrd1p for dislocation, but has no obvious requirements for Ubc6 and the Ubc7p/Cue1p co-factors that normally provide E2 ubiquitin-conjugating activities. This contrasts quite strikingly with a partial requirement for Ubc7/Cue1p and the total lack of requirement for an active Hrd1p for RTA [27]. Although we have not identified any E2 involved (directly or indirectly) for the toxic fraction of SLTxA1, the combined observations point to mechanistic differences between the way these two toxins reach the cytosol.

Since mammalian HRD1 is required for the dislocation of substrates that are not necessarily ubiquitylated [75], [30] the requirement of SLTxA1 for the E3 ligase activity of Hrd1p may be an indirect one, perhaps reflecting auto-ubiquitylation of HRD1/Hrd1p [65] or the turnover of an unknown ER factor that is utilized by the small population of SLTxA1 that can recover activity in the cytosol. Such questions will be addressed in future studies.

## Materials and Methods

### Yeast strains

A yeast gene knockout collection derived from strain BY4741 (MATa *his*Δ1, *leu2*Δ0, *met15*Δ0, *ura3*Δ0) was sourced from Open Biosystems (Huntsville, AL; Table S1). Other strains used are also listed in Table S1. Cultures were grown in rich YPDA media or synthetic media containing standard ingredients and 2% glucose (SD medium), 2% raffinose (SR medium), or 2% galactose +2% raffinose (SRG medium). Where appropriate, media lacking tryptophan, uracil or leucine were utilized. Yeast transformations were performed using the lithium acetate/single-stranded DNA/polyethylene glycol method [76].

### Construction of SLTxA1 clones

The C-terminal region of SLTxA chain is non-covalently associated with a doughnut-shaped pentameric ring of B subunit monomers (SLTxB). During trafficking, a protease-sensitive loop in the C-terminal region of SLTxA is cleaved by furin [31], resulting in a catalytically active SLTxA1 chain disulphide-linked to a small SLTxB-associated A2 fragment. It is the SLTxA1 chain that subsequently dislocates [36]. We therefore truncated the SLTxA gene after the codon expressing arginine 248 to generate a gene encoding SLTxA1 and fused this to the Kar2p signal peptide downstream of the galactose-inducible *GAL1* promoter in a yeast expression vector derived from plasmid pRS316 [77]. Substitution mutants N83Q (to remove an *N*-glycosylation site), and K1R and K11R (to remove canonical ubiquitylation sites) were constructed using a QuickChange II Site-directed mutagenesis kit (Stratagene, La Jolla, USA) using the primers displayed in Table S2.

### Construction of Hrd1 clones

Two approaches were undertaken. In the first, strains were constructed as previously described [64], resulting in expression of HRD1 or *hrd1* variants from the endogenous HRD1 promoter, and integrants were selected on medium lacking tryptophan. In the second approach, HRD1 was amplified from a *S. cerevisiae* genomic library and cloned downstream of a galactose inducible promoter, using standard techniques, in the yeast integrative vector pRS406. Specific mutants were prepared using Quick Change mutagenesis (Stratagene, La Jolla, USA) using the primers displayed in Table S3. The resulting plasmids were transformed into the Open Biosystems *Δhrd1* strain and single integration was confirmed by PCR.

### Pulse-Chase Analysis

Pulse-chase analysis was as previously described, substituting sheep anti-SLTxA antibodies for immunoprecipitation [27]. Products were analyzed by reducing SDS-PAGE and autoradiography and were quantified using TotalLab software (Newcastle upon Tyne, United Kingdom). For the temperature-sensitive CDC48 strain used, scells were grown at the permissive temperature for growth and shifted to the restrictive temperature for 2 h before the start of the radioactive pulse.

### Immunoblotting

Protein samples were separated by SDS-PAGE and immunoblotted. SLTx proteins were identified by serial probing with sheep anti SLTx serum and anti-sheep–alkaline phosphatase conjugate followed by BCIP/NBT color development (Promega, Southampton, UK) and ubiquitin conjugates were identified by serial probing with mouse anti-ubiquitin antibodies (Covance, CA, USA) and anti-mouse peroxidise conjugate and revealed after ECL development (Invitrogen, Paisley, UK).

### Growth Sensitivity to SLTxA1 chain

Yeast cells expressing Kar2p-SLTxA1 were grown overnight in liquid SR medium lacking uracil. Tenfold serial dilutions of cells from 106 to 101 were plated on SG plates lacking uracil to induce expression and on SD plates as controls. Plates were typically incubated at 30°C for 4 d and examined for growth differences. Where only small differences in growth rate were expected, cultures of yeast expressing Kar2p-SLTxA1 were diluted to 4.10^6^ cells.ml^−1^ in both SD and SG liquid medium, growth rate was followed by reading the absorbance (600 nm) of 1∶10 dilutions hourly and doubling times were determined from the exponential phase of growth.

## Supporting Information

Figure S1 **SLTxA1 chain is not** ***N*** **-glycosylated upon ER arrival after mammalian cell challenge.** African green monkey Vero cells cells obtained from ATCC (CCL-81™) were incubated with 1 µg.ml^−1^ SLTx for periods from 15 min up to 240 min. Detergent soluble extracts taken at the times indicated were electrophoresed and immunoblotted. Upper panel: both unprocessed SLTxA1+A2 (grey arrowhead, A1+A2) and furin processed SLTxA1 (black arrowhead, A1) were identified by serial incubation of the blot with sheep anti-SLTx and anti-sheep –alkaline phosphatase conjugate followed by BCIP/NBT color development. Samples of partly-processed SLTxA chain were run in parallel to verify the sizes of SLTx-derived bands obtained from the cell extracts. Offset white arrowheads mark the expected migration positions of *N*-glycosylated SLTxA1+A2 (upper) and *N*-glycosylated SLTxA1 (lower). *, cross-reacting material. Lower panel: the same immunoblot was reprobed for γ-tubulin.
(TIF)

Figure S2 **The bulk population of SLTxA1(N^−^) is polyubiquitylated.** **A.** Immunoprecipitates (anti-SLTx) taken from cell extracts of the drug-sensitive JN284 yeast [81] expressing SLTxA1(N^−^) in the presence or absence of the proteasomal inhibitor clasto-Lactacstin ß-lactone (cLß-l) were separated by SDS-PAGE and after immunoblotting, ubiquitylated SLTxA1 (bar, right hand side of the panel) was identified by serial probing with mouse anti-ubiquitin antibodies and anti-mouse peroxidise conjugate and revealed after ECL development. Positions of migration of molecular weight standards are shown on the left.
(TIF)

Table S1 (DOC)

Table S2 (DOC)

Table S3 (DOC)
