# Supplementary figures and images for: Cytosolic Entry of Shiga-Like Toxin A Chain from the Yeast Endoplasmic Reticulum Requires Catalytically Active Hrd1p
Source: PLoS One. 2012 Jul 19;7(7):e41119. doi: 10.1371/journal.pone.0041119 (PMC3400632; doi:10.1371/journal.pone.0041119)

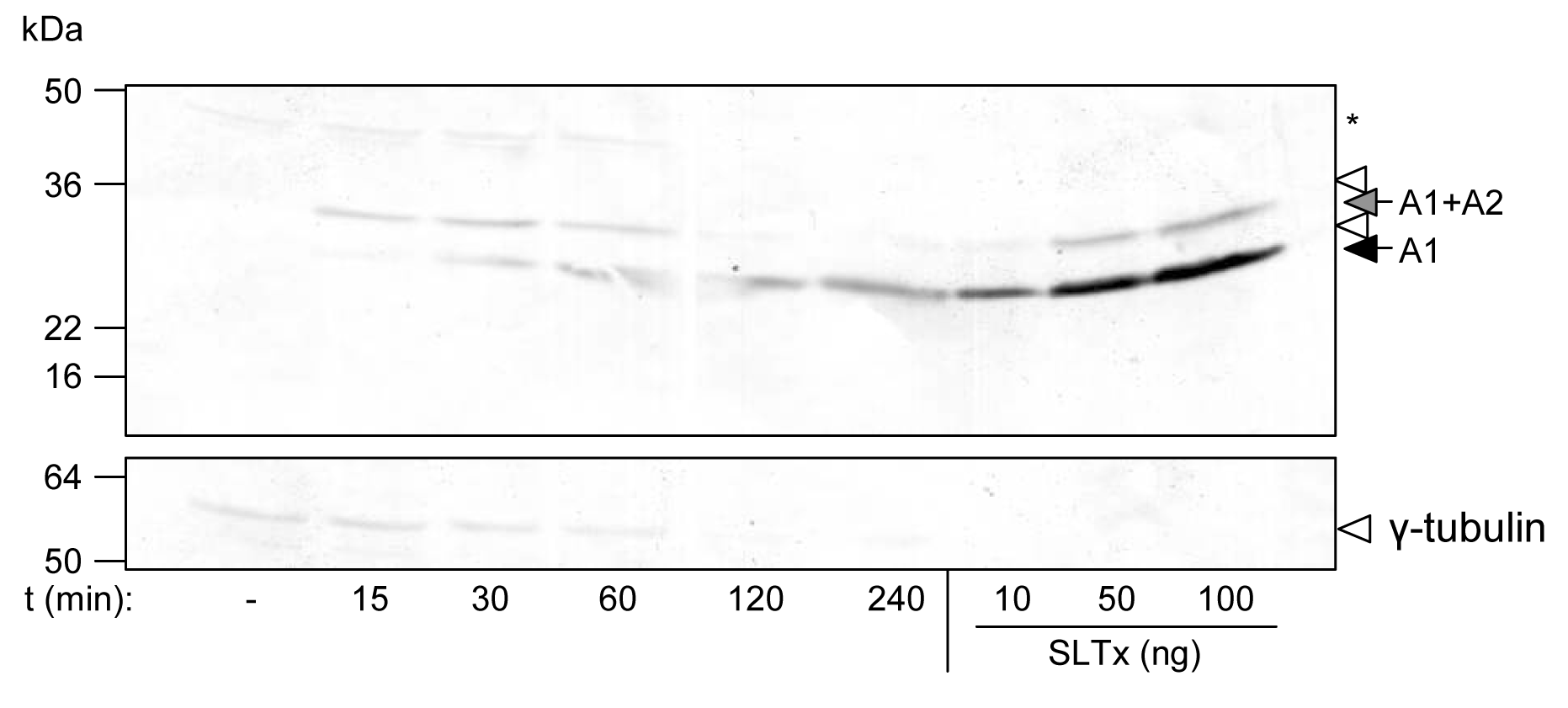

Supplement: Figure S1 — SLTxA1 chain is not N -glycosylated upon ER arrival after mammalian cell challenge. African green monkey Vero cells cells obtained from ATCC (CCL-81™) were incubated with 1 µg.ml−1 SLTx for periods from 15 min up to 240 min. Detergent soluble extracts taken at the times indicated were electrophoresed and immunoblotted. Upper panel: both unprocessed SLTxA1+A2 (grey arrowhead, A1+A2) and furin processed SLTxA1 (black arrowhead, A1) were identified by serial incubation of the blot with sheep anti-SLTx and anti-sheep –alkaline phosphatase conjugate followed by BCIP/NBT color development. Samples of partly-processed SLTxA chain were run in parallel to verify the sizes of SLTx-derived bands obtained from the cell extracts. Offset white arrowheads mark the expected migration positions of N-glycosylated SLTxA1+A2 (upper) and N-glycosylated SLTxA1 (lower). *, cross-reacting material. Lower panel: the same immunoblot was reprobed for γ-tubulin. (TIF) [file pone.0041119.s001.tif]

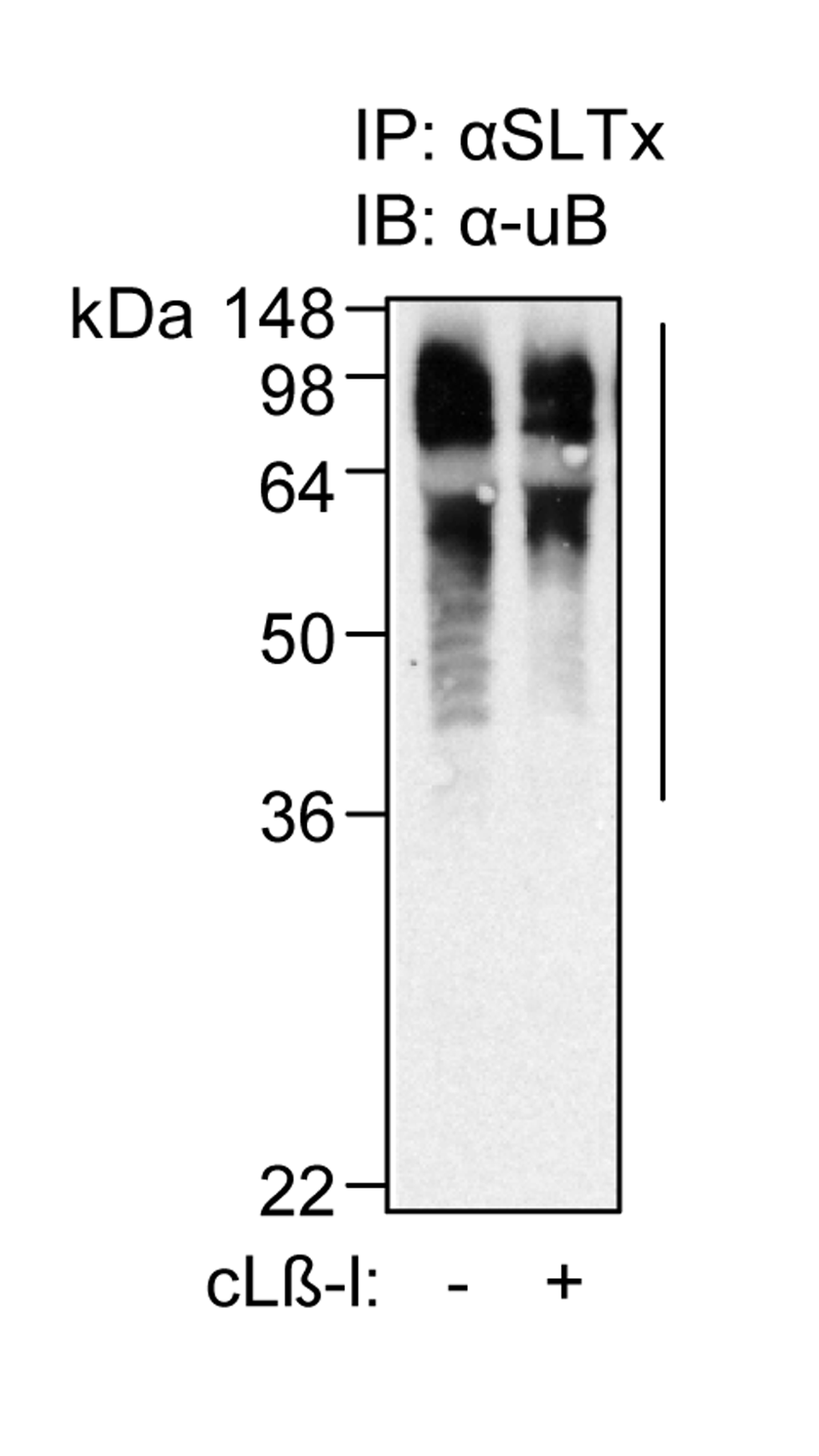

Supplement: Figure S2 — The bulk population of SLTxA1(N−) is polyubiquitylated. A. Immunoprecipitates (anti-SLTx) taken from cell extracts of the drug-sensitive JN284 yeast [81] expressing SLTxA1(N−) in the presence or absence of the proteasomal inhibitor clasto-Lactacstin ß-lactone (cLß-l) were separated by SDS-PAGE and after immunoblotting, ubiquitylated SLTxA1 (bar, right hand side of the panel) was identified by serial probing with mouse anti-ubiquitin antibodies and anti-mouse peroxidise conjugate and revealed after ECL development. Positions of migration of molecular weight standards are shown on the left. (TIF) [file pone.0041119.s002.tif]
