# Supplementary material for: Cytosolic Entry of Shiga-Like Toxin A Chain from the Yeast Endoplasmic Reticulum Requires Catalytically Active Hrd1p
Source: PLoS One. 2012 Jul 19;7(7):e41119. doi: 10.1371/journal.pone.0041119 (PMC3400632; doi:10.1371/journal.pone.0041119)
Supplement: Table S1 — (DOC) [file pone.0041119.s003.doc]

| strain | Genotype or relevant features | Source or  reference |
| --- | --- | --- |
| BY4741 | MATa *his*∆*1*, *leu2*∆*0*, *met15*∆*0*, *ura3*∆*0* | Open Biosystems |
| Single gene knockout library members | *BY4741, Δpmt1*, *Δpmt2, Δpmt3, Δpmt5, Δrad23, Δpng1, Δbst1, Δpep4, Δhrd1, Δhrd3, Δusa1, Δder1, Δerp1, Δerp2, Δerp3, Δerp4, Δerp5, Δerp6, Δemp24, Δerv25, as appropriate* | Open Biosystems |
| *cdc48-1* | *cdc48-1, prc1-1, ura3-52, ade2-101, lys2-801, can1-100* |  |
| Cl3-ABYS-86 | MATα *pra1-1 prb1-1 prc1-1 cps1-3 ura3*-*5 leu2-3, canR* |  |
| *pre1-1* | Cl3-ABYS-86*, pre1-1* |  |
| *pre2-2* | Cl3-ABYS-86*, pre2-2* |  |
| JN284 | MATα ura3-52, leu2 (-3,-112), his7-2, ise1 |  |
| YTX005 | MATα *Δubc6::HIS3, trp1-1, his3-Δ200, ura3-52, lys2-801, leu2(-3, -112)* |  |
| YTZ106 | MATα *Δubc7::LEU2, trp1-1(am), his3-Δ200, ura3-52, lys2-801, leu2(-3, -112)* |  |
| YTX135 | MATα *Δubc7::LEU2,* *Δubc6::HIS3*, *trp1-1(am), his3-Δ200, ura3-52, lys2-801, leu2(-3, -112)* |  |
| RHY3011 | *ade2, met2, lys2-801, ura3-52, HMG1, HMG2, trp1::hisG, leu2Δ, his3Δ200, hrd1Δ::KanMX* |  |
| RHY3011 derivatives | RHY3011 expressing HRD1 or different *hrd1* variants | and this study |
