# Supplementary material for: Cytosolic Entry of Shiga-Like Toxin A Chain from the Yeast Endoplasmic Reticulum Requires Catalytically Active Hrd1p
Source: PLoS One. 2012 Jul 19;7(7):e41119. doi: 10.1371/journal.pone.0041119 (PMC3400632; doi:10.1371/journal.pone.0041119)
Supplement: Table S2 — (DOC) [file pone.0041119.s004.doc]

| **SLTxA1 N83Q (N-)** |  |
| --- | --- |
| QCSLTN83QF | GTGACAGGATTTGTTCAAAGGACAAATAATG |
| QCSLTN83QR | CATTATTTGTCCTTTGAACAAATCCTGTCAC |
| **SLTxA1(N-) K1R** |  |
| QCSLT1KRF | gttaGAGGTGCCGATGATAGGGAATTTACCTTAGacttc |
| QCSLT1KRR | GAAGTctaaggtaaattccctatcatcggcacctcTAAC |
| **SLTxA1(N-) K11R** |  |
| QCSLT11KRF | GACTTCTCGACTGCAAgGACGTATGTAGATTC |
| qcslt11krr | gaatctacatacgtccttgcagtcgagaagtc |
