# Supplementary material for: Cytosolic Entry of Shiga-Like Toxin A Chain from the Yeast Endoplasmic Reticulum Requires Catalytically Active Hrd1p
Source: PLoS One. 2012 Jul 19;7(7):e41119. doi: 10.1371/journal.pone.0041119 (PMC3400632; doi:10.1371/journal.pone.0041119)
Supplement: Table S3 — (DOC) [file pone.0041119.s005.doc]

| **HRD1 L61A** |  |
| --- | --- |
| QCHRD1FL61A | CTTATTAAATTCTACCGCACTATGGCAACTCCTAAC |
| QCHRD1RL61A | GTTAGGAGT TGCCATAGTGCGGTAGAATTTAATAAG |
| **HRD1 L74A** |  |
| QCHRD1FL74A | CTATTATTTGGTGAAGCGAGGCTTATTGAGCATG |
| QCHRD1RL74A | CATGCTCAATAAGCCTCGCTTCACCAAATAATAG |
| **HRD1 E78A** |  |
| QCHRD1FE78A | GAACTGAGGCTTATTGCGCATGAGCACATTTTTG |
| QCHRD1RE78A | CAAAAATGTGCTCATGCGCAATAAGCCTCAGTTC |
| **HRD1 S9898A** |  |
| QCHRD1FS9798A | CACCTTGTTTATGGCCGCACTGTTCCACGAAC |
| QCHRD1FS9798A | GTTCGTGGAACAGTGCGGCCATAAACAAGG TG |
| **HRD1 W123A** |  |
| QCHRD1FW123A | CTATCTGAAAGTTTTCCATGCGATTTTAAAGGATAGGCT |
| QCHRD1RW123A | CAGCCTATCCTTTAAAATCGCATGGAAAACTTTCAGATAG |
| **HRD1 L209A** |  |
| QCHRD1L209A | CCTACAGACTTGTCGGAATTTCTGGGAATTTTATC |
| QCHRD1L209A | GATAAAATTCCCAGAAATTCCGACAAGTCTGTAGG |
